# Environmental Glyphosate Exposure Is Associated with Lower Serum 25-Hydroxyvitamin D Concentrations: A Cross-Sectional Analysis of NHANES 2013–2018

**DOI:** 10.3390/biology15171510

**Published:** 2026-09-03

**Authors:** Zsolt Gáll, Lénárd Farczádi, Melinda Kolcsar

**Affiliations:** 1 Department of Pharmacology and Clinical Pharmacy, George Emil Palade University of Medicine, Pharmacy, Science and Technology of Targu Mures, Gheorghe Marinescu Street, No. 38, 540142 Targu Mures, Romania; melinda.kolcsar@umfst.ro; 2 Scidata Research and Training Ltd., 547367 Targu Mures, Romania; 3 Chromatography and Mass Spectrometry Laboratory, Center for Advanced Medical and Pharmaceutical Research, George Emil Palade University of Medicine, Pharmacy, Science and Technology of Targu Mures, 540142 Targu Mures, Romania; lenard.farczadi@umfst.ro

**Keywords:** glyphosate, herbicide, vitamin D, vitamin D deficiency, nutrition surveys, NHANES

## Abstract

Glyphosate is an herbicide used to control plant growth and ripen specific crops. Glyphosate exposure has been associated with cancer, metabolic and neurological diseases, and reproductive issues in humans. A common element linking these disorders could be vitamin D deficiency. However, the relationship between glyphosate exposure and vitamin D status in the general population remains unclear. This analysis focused on the association between urinary glyphosate levels and serum 25-hydroxy vitamin D [25(OH)D] concentrations in a large representative sample of U.S. adults. Data from 4284 participants in the National Health and Nutrition Examination Survey (NHANES) between 2013–2018 were analyzed. Serum 25(OH)D levels were modeled using multivariable weighted linear regression, with adjustment for age, sex, race/ethnicity, body mass index, season, education, income, physical activity, smoking, and kidney function. Urinary glyphosate concentration was inversely associated with serum 25(OH)D in both the design-based univariate weighted analysis (β = −3.537, *p* = 0.035) and adjusted survey-weighted models for major confounding factors (β = −4.208, *p* = 0.009), using Taylor linearization for variance estimation to account for the complex NHANES survey design. In stratified analysis, the relationship between total vitamin D intake and serum 25(OH)D was found to be significantly modified by glyphosate exposure, with stronger dietary dependence observed in the high-exposure tertile (β = 12.88, *p* < 0.0066) than in the low-exposure tertile (β = 7.41, *p* < 0.0165). Higher urinary glyphosate concentration was associated with lower serum 25(OH)D levels in U.S. adults, suggesting it may represent an unrecognized risk factor for vitamin D deficiency. Longitudinal and experimental studies are needed to clarify causality, the underlying mechanisms, and public health implications.

## Simple Summary

Vitamin D is essential for bone health and may also support normal immune, muscle, and metabolic function. Many adults have low vitamin D levels, but the reasons are not fully understood. Glyphosate is a widely used herbicide that people may be exposed to through food, water, or their environment. We examined whether glyphosate exposure was related to vitamin D levels in a nationally representative sample of United States adults. People with higher glyphosate concentrations in urine tended to have lower concentrations of vitamin D in their blood, even after accounting for factors known to influence vitamin D, including age, sex, body weight, season, diet, physical activity, income, and kidney function. We also found that, among participants with higher glyphosate exposure, blood vitamin D levels appeared to depend more strongly on vitamin D obtained from diet and supplements. Because this study measured exposure and vitamin D at one point in time, it cannot prove that glyphosate causes lower vitamin D levels. Nevertheless, the findings identify a possible environmental contributor to poor vitamin D status and support further prospective and experimental studies to determine whether the association is causal and to identify potential biological mechanisms.

## 1. Introduction

Glyphosate is an organophosphorus compound used as an herbicide to control plant growth and ripen specific crops. It has become one of the most widely used pesticides globally since its introduction in 1974 [1]. By inhibiting the shikimate pathway, a critical metabolic route found in plants and microorganisms but absent in humans and animals, it selectively targets the unwanted vegetation. Although its toxicity has been assessed multiple times by regulatory authorities and an acceptable daily intake was established, emerging epidemiological data showed that exposure to glyphosate was associated with certain types of cancer, cardiovascular, metabolic, and neurological diseases [2,3,4,5]. In addition, experimental studies have identified glyphosate-related disruptions in mineral metabolism, endocrine system, and mitochondrial function at very low doses [6]. Human exposure to glyphosate includes various potential sources such as surface water, atmospheric air, contaminated soil, and foods [7]. Although occupational exposure remains the most important health risk, an increasing trend has also been reported in the general population over the last two decades [8]. Collectively, these findings suggest that the health consequences of this widespread environmental exposure are not yet fully understood, and glyphosate may possess complex biological effects that remain unrevealed in the general population.

Given the multifaceted role of vitamin D—which extends far beyond bones and minerals, including metabolic, inflammatory, and neurological processes [9,10,11]—the effects of glyphosate on the endogenous synthesis, exogenous intake, or activation of vitamin D may be involved in the etiopathogenesis of many of the previously mentioned disorders. Epidemiological studies have consistently documented associations between vitamin D deficiency and increased risk of autoimmune diseases, infections [12], cardiovascular disease [13], metabolic syndrome [14], certain malignancies [15], and neurological and psychiatric disorders [16,17]. The high prevalence of vitamin D deficiency in developed countries triggered intensive research into modifiable environmental and dietary determinants of vitamin D status [18].

Vitamin D is synthesized in skin by transforming a precursor (7-dehydrocholesterol) under UVB sunlight exposure to cholecalciferol (vitamin D3) or ingested with food as cholecalciferol or ergocalciferol (vitamin D2), which are further metabolized by a series of enzymes belonging to the cytochrome P450 (CYP) family. The conversion of dietary calciferols to 25-hydroxy vitamin D [25(OH)D] is catalyzed primarily by CYP2R1 and CYP27A1 in the liver, while the subsequent activation of 25(OH)D to its active metabolite 1,25-dihydroxy vitamin D [1,25(OH)_2_D] involves CYP27B1 (1α-hydroxylase) expressed in the kidney and other tissues [11]. The catabolism and inactivation of vitamin D metabolites is mediated by hydroxylation at C24 catalyzed by the CYP24A1 enzyme [19]. Many other CYP enzymes are involved in the transformation of vitamin D precursors and metabolites [20], including CYP11A1, which produces metabolites with non-calcemic effects, particularly anti-inflammatory, anti-cancer, and pro-differentiation properties [21]. Because vitamin D hydroxylation involves cytochrome P450 enzymes, experimental reports of altered CYP activity following glyphosate-based herbicide exposure raise a hypothesis that glyphosate exposure could be related to vitamin D metabolism [22,23,24,25,26,27]. However, whether such mechanisms occur at environmental human exposure levels remains unknown.

To date, no large-scale epidemiological studies have systematically examined the relationship between environmental glyphosate exposure and vitamin D status in the general population. Previous studies have focused on organochlorine compounds [28,29] or have examined genetic variants in the vitamin D pathway without direct assessment of environmental exposures. On the other hand, several recent studies leveraged data from the National Health and Nutrition Examination Survey (NHANES), a nationally representative survey that included urinary glyphosate measurements to show that glyphosate exposure influences glucose homeostasis [30], cognitive function, increases the risk of depression, neurological diseases [31], and inflammatory conditions [4], which might also be related to reduced vitamin D levels.

The present analysis was designed to fill this critical gap by using serum 25(OH)D and urinary glyphosate levels by combining three cycles of NHANES data, including a large sample of U.S. adults. The main hypothesis was that environmental glyphosate exposure would be inversely associated with serum 25(OH)D concentrations and that this association would persist after adjustment for established determinants of vitamin D status, including age, season, body composition, skin pigmentation, dietary and supplementary intake, and physical activity.

## 2. Materials and Methods

### 2.1. Study Design

#### 2.1.1. Subjects

Data were obtained from the National Health and Nutrition Examination Survey (NHANES), a program of the US Centers for Disease Control and Prevention (CDC) that employs stratified multistage sampling to collect nationally representative health and laboratory data. Anonymized data are publicly available at https://www.cdc.gov/nchs/nhanes/ (accessed on 27 November 2025). NHANES was used because it provides individual-level linkage of urinary glyphosate measurements, serum 25(OH)D, dietary and supplement information, relevant covariates, and survey-design variables. Urinary glyphosate concentrations were measured across three consecutive two-year NHANES cycles spanning 2013 to 2018, as these represent the only cycles in which glyphosate was included in the NHANES environmental chemical panel. Serum 25(OH)D concentration, used to assess vitamin D status, is included as a standard laboratory measure in every NHANES cycle and is thus available across all survey years. The following exclusion criteria were applied sequentially to obtain the final analytical sample: missing laboratory data for urinary glyphosate concentration, serum 25(OH)D concentration, serum creatinine, missing anthropometric or demographic data, children, and pregnant women.

#### 2.1.2. Variables

Urinary glyphosate and serum 25(OH)D concentrations were the primary variables of interest. Glyphosate was measured using 2D ion chromatography-tandem mass spectrometry (IC-MS/MS) with isotope dilution quantification [32]. Urine samples were collected and processed according to standardized protocols: samples were aliquoted within 4 h of collection and frozen at −20 °C for transport to the CDC’s National Center for Environmental Health laboratory. The analytical method achieved a lower limit of detection (LOD) of approximately 0.1 ng/mL, with quality control procedures including replicate analyses, blank samples, and reference materials analyzed with each batch to ensure accuracy and precision. For analytes with results below the LOD, urinary glyphosate concentration was imputed by dividing the LOD by the square root of 2. Urinary glyphosate was analyzed primarily as log10-transformed concentration in µg/L. Because spot urine concentration may vary with hydration and urine flow, urinary creatinine was included as a continuous covariate in a prespecified sensitivity model. This regression-based approach was selected to assess dilution-related confounding without relying solely on a glyphosate-to-creatinine ratio [33,34].

Serum 25(OH)D was used as the indicator of vitamin D status. For descriptive interpretation, vitamin D deficiency was defined as serum 25(OH)D < 50 nmol/L (<20 ng/mL), insufficiency as 50–74.9 nmol/L (20–29.9 ng/mL), and sufficiency as ≥75 nmol/L (≥30 ng/mL) [35]. Serum 25(OH)D was analyzed as a continuous variable in the primary regression models. Serum 25(OH)D concentrations were determined using high-performance liquid chromatography-tandem mass spectrometry (HPLC-MS/MS) for the quantitative detection of 25-hydroxyvitamin D3 [25(OH)D3], 3-epi-25-hydroxyvitamin D3 [epi-25(OH)D3], and 25-hydroxyvitamin D2 [25(OH)D2] in human serum as reported in previous studies. The detailed description of the analytical method and the observed trends throughout the last two decades were summarized recently [36].

Covariates were identified based on prior epidemiological studies and NHANES analyses demonstrating associations with vitamin D status and glyphosate exposure: age, sex, race/ethnicity, education, income-to-poverty ratio, body mass index (BMI), examination season, alcohol use, smoking, and physical activity. Estimated glomerular filtration rate (eGFR), calculated using the CKD-EPI 2021 equation, was also included to adjust for urinary glyphosate variation related to renal function. Multicollinearity among covariates was checked by computing survey-weighted variance inflation factors (VIF) for all predictors, including sets of indicator variables for categorical covariates, using the weighted least squares design; all variables with VIF <5 were included.

All covariates were transformed into categorical variables. Age, sex, and race categories were derived from NHANES demographic data, whereas education, BMI, smoking, alcohol consumption, income-to-poverty ratio, season, and physical activity categories were created or adapted by the authors for analytical uniformity and interpretability. Age was divided into three groups: 18–39, 40–59, and over 60 years. Sex was defined using the NHANES demographic variable recorded as male or female. No gender identity measure was available in the dataset; therefore, all analyses refer to sex rather than gender. Race was collapsed into four categories as in previously published studies: Non-Hispanic White, Hispanic including Mexican Americans, Non-Hispanic Black, and Other Race [37,38]. Education, originally reported by NHANES as five levels (“Less than 9th grade,” “9–11th grade,” “High school graduate/GED,” “Some college or AA degree,” “College graduate or above”), was merged by the authors into two categories to reflect general educational attainment: “High school diploma or less”—first three NHANES levels—and “College and above”—last two levels. BMI categories followed clinical standards, with normal weight defined as <25 kg/m^2^, overweight as 25–29.9 kg/m^2^, and obesity as ≥30 kg/m^2^. Smoking status used the NHANES criterion of lifetime cigarette consumption: individuals who reported smoking ≥100 cigarettes were classified as “yes,” while those who had not were classified as “no”. Alcohol consumption, derived from NHANES questionnaire responses, was dichotomized by the authors into “Abstainer/Light consumer” (<2 drinks/day) and “Moderate/Heavy consumer” (≥2 drinks/day) to differentiate low- and high-risk drinking behaviors. The income-to-poverty ratio, a NHANES variable, was categorized by the authors into low-income (<1.3), middle-income (≥1.3–<3.5), and high-income (≥3.5) groups following U.S. federal assistance thresholds. The season of examination, recorded by NHANES, was classified as “Winter” (November–April) or “Summer” (May–October) to account for potential seasonal variation in vitamin D. Physical activity was categorized by the authors into “Active” and “Inactive” based on responses to NHANES questions about moderate and vigorous recreational activity; participants reporting no such activity were designated as “Inactive”.

Daily dietary and supplemental vitamin D intake were considered as main predictors of serum 25(OH)D and were included in regression analysis. Dietary vitamin D intake was obtained from two 24-h dietary recall interviews conducted by trained nutritionists. The first interview was administered in-person, while the second was conducted via phone call 3–10 days later. The interview collected detailed information on all foods and beverages consumed in the previous 24 h, including portion sizes and preparation methods. Dietary vitamin D intake was calculated from the NHANES Food and Nutrient Database using the U.S. Department of Agriculture nutrient composition data. Total dietary vitamin D intake, expressed as micrograms per day, was calculated as the average of the two 24-h recalls and included both naturally occurring and fortified food sources. Supplemental vitamin D intake was obtained from the two dietary supplement-use questionnaires administered during the interviews. Participants reported the name, frequency, and dose of all vitamin D-containing supplements taken in the past month. Vitamin D supplement dose was calculated based on reported frequency of use and documented label dose. Participants who did not report use of any vitamin D-containing supplement were assigned a supplemental intake of 0 μg/day, consistent with standard NHANES dietary supplement use conventions. Total vitamin D intake was calculated by summing the dietary and supplemental intakes, with the latter treated as 0 μg/day for non-users. Descriptive statistics for supplemental vitamin D intake were restricted to participants who reported any supplement use, to characterize dose among users; in contrast, total vitamin D intake used in regression models reflects the full analytic sample, incorporating a value of 0 μg/day for non-users.

### 2.2. Regression Analysis

NHANES applied a complex, multistage probability sampling design, which includes stratification, clustering, and sampling weights to maintain representativeness. Based on NHANES recommendations, this study used the survey design variables for strata, primary sampling units, and the specific subsample weights corresponding to the urinary glyphosate investigation throughout all statistical analyses. To combine the three two-year NHANES cycles (2013–2014, 2015–2016, and 2017–2018), the cycle-specific glyphosate subsample weight was divided by three to construct a pooled six-year weight. Survey-weighted linear regression models with Gaussian distribution and identity link were fitted, and standard errors, 95% confidence intervals, and hypothesis tests were estimated using Taylor-series linearization to account for the complex sampling design.

Since normality tests showed that urinary glyphosate concentration showed a lognormal distribution, it was log-transformed (base 10) for all subsequent analyses. To evaluate the association between urinary glyphosate concentration and serum 25(OH)D levels, sequential models were fitted including univariable (Model 1) and multivariable models accounting for basic demographic covariates such as age, sex, race/ethnicity, and BMI (Model 2), as well as socioeconomic and lifestyle factors including education, income, smoking, alcohol consumption, and physical activity (Model 3). Finally, renal function, as a crucial influencing factor of urinary glyphosate concentration, was also considered (Model 4). No imputation was applied; all models used complete case data only. Participants with missing data on the outcome, exposure, or any covariate included in a given model were excluded from that model. The baseline analytic sample comprised 4284 participants with complete urinary glyphosate, serum 25(OH)D, and core demographic data. Further reduction of the sample was required by missing dietary vitamin D intake, yielding 3997 participants; 287 participants (6.7%) had missing dietary vitamin D intake. The fully adjusted model included 3645 participants. The further reduction of 352 participants was driven primarily by missing income-to-poverty ratio (*n* = 350), with one participant each missing urinary creatinine and smoking-status data. The best model was chosen based on Akaike Information Criterion (AIC) values. Total vitamin D intake was standardized by subtracting the sample mean and dividing by the sample standard deviation, so that regression coefficients represent the change in serum 25(OH)D per 1-SD higher intake. This parameter was included in all multivariate models. Residual diagnostics were performed to assess model assumptions. Sensitivity analyses included log-transformed vitamin D as the outcome variable. Restricted cubic splines analysis was not performed due to sample size limitations within covariate strata, but sensitivity analyses using categorical tertiles and alternative log-transformations were implemented. To evaluate the potential influence of urinary dilution, a sensitivity model replaced CKD stage with urinary creatinine, included as a standardized continuous covariate, while retaining log10-transformed urinary glyphosate as the exposure. This regression-based approach evaluates whether the glyphosate–25(OH)D association is sensitive to adjustment for urine concentration.

Statistical analyses were performed using Python version 3.12.7 (packages: pandas, numpy, scipy, statsmodels, svy). Linear trend tests were conducted across glyphosate exposure tertiles using weighted regression. Two-tailed statistical significance was set at α = 0.05, and 95% confidence intervals (CI) were calculated for all coefficients.

## 3. Results

**Figure 1:**
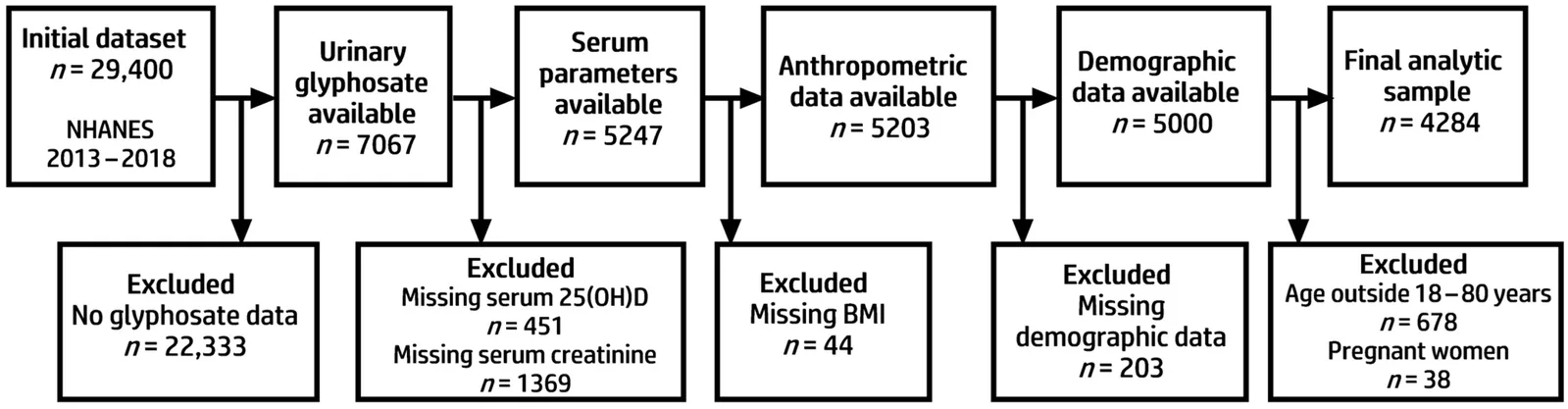
Flowchart of participant selection from the National Health and Nutrition Examination Survey (NHANES) 2013–2018.

Of 29,400 participants from three merged cycles, 7067 (24%) had available urinary glyphosate measurements and were eligible for initial assessment. After sequential exclusions for missing serum 25(OH)D (*n* = 451), missing serum creatinine (*n* = 1369), missing BMI (*n* = 44), missing demographic data (*n* = 203), age outside 18–80 years (*n* = 678), and pregnancy (*n* = 38), a final sample of 4284 participants was included in the analysis (Figure 1).

Analyses were weighted according to NHANES protocols to produce population-representative estimates rather than sample-based associations. The 4284 participants represented 222,906,724 subjects (weighted). The weighted cohort included 48.6% males with a mean (SD) age of 48.0 (17.1) years, having an average BMI of 29.4 (7.13). Table 1 summarizes the distribution of demographic, socio-economic and other characteristics by tertile of urinary glyphosate concentration. The distribution of participants differed significantly by age (*p* < 0.0001), sex (*p* = 0.0003), race/ethnicity (*p* < 0.05), BMI (*p* < 0.0001), and income-to-poverty ratio (*p* = 0.0002). Specifically, higher glyphosate exposure was associated with male sex, Hispanic American or Non-Hispanic Black identification, higher BMI categories, and lower income. Dietary vitamin D intake did not differ across glyphosate-exposure tertiles (*p* = 0.712). Supplement use showed no statistically significant trend across tertiles (35.6%, 34.5%, and 32.7%, respectively; *p* = 0.098), and the dose of supplemental vitamin D among users also did not differ significantly (*p* = 0.091). Current smoking status (*p* = 0.159), education level (*p* = 0.272), alcohol consumption (*p* = 0.847), and recreational physical activity (*p* = 0.178) also did not differ significantly across glyphosate exposure tertiles.

Multicollinearity was assessed using VIF on the weighted design matrix. Results are presented in Supplementary Table S1. All covariates showed acceptable VIF values (range: 1.02–6.03, mean: 2.35). The highest VIF was observed for the Non-Hispanic White race category (VIF = 6.03), which is expected for categorical dummy variables and reflects inherent correlation between mutually exclusive race categories rather than problematic multicollinearity. No variables exceeded the conventional threshold of VIF > 10, and the adjustment set was retained without modification. All mean VIF values calculated by categorical variables were below 5 (Supplementary Table S1).

The univariate survey-weighted regression model revealed a significant inverse association (β = −3.537, *p* = 0.035) between serum 25(OH)D levels and glyphosate exposure expressed as log-transformed urinary glyphosate concentrations, meaning that a 10-fold increase in urinary glyphosate concentration corresponded to a 3.54 nmol/L decrease in serum 25(OH)D. Multivariate models were constructed by sequentially controlling for previously demonstrated covariates, and the obtained AIC values showed that the fully adjusted model (Model 4) was the most appropriate (Table 2). The analytic sample sizes varied by model because complete-case analysis was applied according to the covariates included. The final CKD-adjusted survey-weighted model included 3645 participants.

The multivariate analysis, including all covariates, demonstrated that glyphosate exposure remained significantly associated with lower serum 25(OH)D (t = −2.879, *p* = 0.0085, Table 3). As expected, the dominant predictor was total vitamin D intake in the fully adjusted model (t = 2.645, *p* < 0.0145). In addition, older age (t = 8.976, *p* < 0.0001), male sex (t = −6.024, *p* < 0.0001), race (t = 8.788, *p* < 0.0001), obesity (t = −7.159, *p* < 0.0001), higher income-to-poverty ratio (t = 3.121, *p* = 0.0048), winter season (t = −3.634, *p* = 0.0014), and inactive lifestyle (t = −4.328, *p* < 0.001) were significant covariates of serum 25(OH)D levels. However, education level, smoking, and alcohol consumption had not significantly impacted serum 25(OH)D levels.

**Figure 2:**
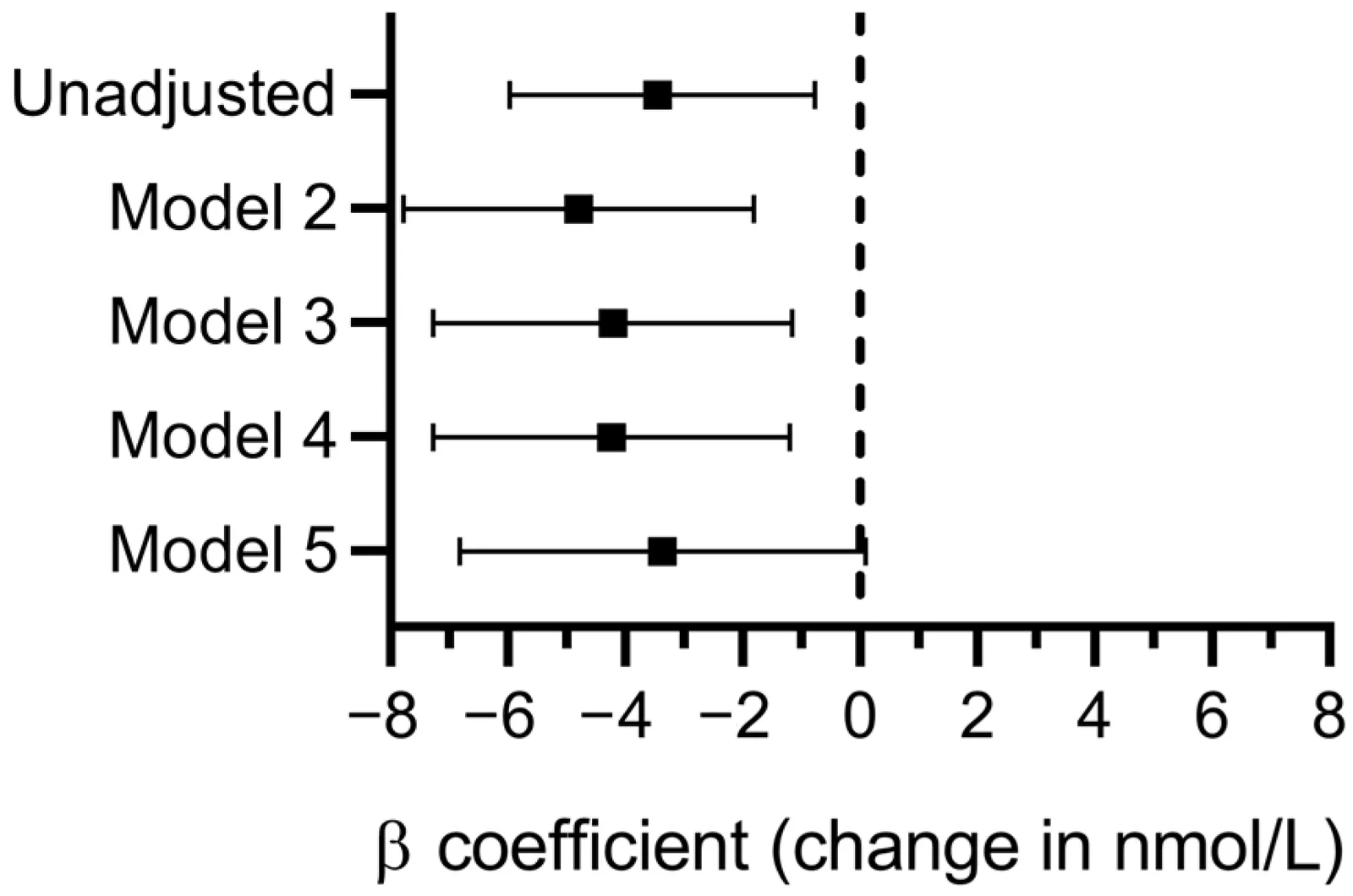
Forest plot displaying beta coefficients and 95% confidence intervals for the association between urinary glyphosate and serum 25(OH)D across unadjusted, adjusted, and sensitivity models. The vertical dashed line at 0 indicates the line of no effect.

To assess robustness to the non-normality observed in residuals of the linear-log model, a sensitivity analysis was conducted using log-transformed serum 25(OH)D values. The association between glyphosate exposure and vitamin D status persisted with similar magnitude and significance (β = −0.0294, *p* = 0.0034), corresponding to a 2.9% decrease in serum 25(OH)D per 10-fold increase in urinary glyphosate (Figure 2). The two models showed nearly identical R^2^ values (0.312 vs. 0.308, Supplementary Table S2). These results confirm that the primary findings are not artefacts of the distributional assumptions.

A stratified analysis was performed across tertiles of urinary glyphosate concentration. The distribution of glyphosate exposure within each tertile was as follows: low exposure (Q1) = 0.071–0.216, medium exposure (Q2) = 0.217–0.455, and high exposure (Q3) = 0.456–8.21. The fully adjusted multivariable survey-weighted regression model using Taylor linearization for variance estimation was re-fitted within each tertile, with all covariates retained in each stratum. Model fit was comparable across all three tertiles (R^2^ range: 0.312–0.335), indicating stable model performance and consistent confounder adjustment (Supplementary Table S3).

In these stratified models, most of the studied covariates showed consistent associations with serum 25(OH)D across glyphosate exposure tertiles. Total vitamin D intake was significantly associated with serum 25(OH)D in low and high glyphosate exposure tertiles (*p* = 0.0165 and *p* = 0.0066, respectively). However, the magnitude of this association differed substantially across glyphosate exposure levels. In the low-glyphosate tertile, each 1 SD increase in vitamin D intake corresponded to a 7.41 nmol/L increase in serum 25(OH)D (95% CI: 1.48–13.34). In the medium-glyphosate tertile, this association was attenuated and became non-significant. Conversely, in the high-glyphosate tertile, the association was substantially amplified: each 1 SD increase in vitamin D intake was associated with a 12.88 nmol/L increase in serum 25(OH)D (95% CI: 3.93–21.83), a 74% increase relative to the low-exposure stratum (Supplementary Table S3). In addition, obesity was a strong negative predictor in all strata (β range −8.0 to −9.4 nmol/L vs. normal BMI, all *p* < 0.001). As expected, Non-Hispanic Black race, male sex, winter season, and physical inactivity were generally associated with lower serum 25(OH)D, whereas higher income and Non-Hispanic White race were associated with higher concentrations, with similar directions of effect across tertiles.

The observed difference in the association between total vitamin D intake and serum 25(OH)D across glyphosate-exposure tertiles is an exploratory finding. Mean total vitamin D intake did not differ significantly across glyphosate tertiles (low: 21.63 ± 69.59 μg/day; medium: 20.32 ± 68.75 μg/day; high: 20.05 ± 63.37 μg/day; *p* = 0.682), indicating that dietary intake alone cannot explain the elevated intake-to-serum coefficient in the high-exposure group. Instead, the amplified dietary response in high-glyphosate-exposure individuals suggests enhanced reliance on dietary vitamin D sources. Mean serum 25(OH)D concentrations trended lower in the high-glyphosate tertile (65.38 ± 28.55 nmol/L) compared to the low tertile (67.26 ± 29.01 nmol/L; *p* = 0.011), further supporting that higher glyphosate exposure levels were associated with lower serum 25(OH)D levels.

## 4. Discussion

This analysis represents the first large-scale epidemiological investigation of the association between environmental glyphosate exposure and serum 25(OH)D concentrations in a nationally representative sample of U.S. adults. The main finding was the inverse association between urinary glyphosate concentration and serum 25(OH)D levels in both univariable and multivariable-adjusted models, corresponding to a 4.19 nmol/L decrease in serum vitamin D per 10-fold increase in urinary glyphosate. While modest in absolute terms, this effect is clinically meaningful in the context of population-level vitamin D deficiency, the prevalence of vitamin D insufficiency in the U.S. population being approximately 40% [39,40]. Given this widespread baseline deficiency, even modest reductions in serum 25(OH)D levels that can be attributed to modifiable environmental exposures are of public health importance.

The survey weighted regression models identified established determinants of vitamin D status, including total vitamin D intake as the dominant predictor, along with age, male sex, winter season, obesity, and physical inactivity. These associations align with published literature and provide internal validation of model adequacy [41,42,43,44].

However, the relationship between eGFR and serum 25(OH)D is causing controversy in clinical research. While many studies reported that low serum 25(OH)D was associated with lower eGFR, i.e., worse kidney function [45], recent evidence showed reduced 25(OH)D clearance in advanced kidney disease [46]. Nevertheless, the adjusted regression models showed a positive association between serum 25(OH)D and eGFR, but the interpretation should be cautious, given that 93.3% of participants had normal eGFR and only 0.8% had advanced CKD. This imbalance may limit the stability of estimates for CKD subgroups.

Interestingly, smoking was not a significant predictor of serum 25(OH)D in the current analysis, despite evidence from prior studies suggesting that smokers are likely to have lower circulating vitamin D levels [47,48]. However, the inverse association between urinary glyphosate and serum 25(OH)D persisted after adjustment for measured dietary intake, supplemental intake, and established demographic, seasonal, lifestyle, socioeconomic, and renal determinants of vitamin D status.

Furthermore, the stratified analysis revealed a non-linear pattern in which the relationship between total vitamin D intake and serum 25(OH)D concentrations was amplified in the high-exposure glyphosate tertile compared to the low-exposure tertile. This increase in the association between intake and serum concentration suggests that individuals with high glyphosate exposure may be more dependent on dietary vitamin D sources to maintain adequate serum concentrations.

The biological mechanisms underlying the observed association cannot be determined from this cross-sectional analysis. Vitamin D status reflects cutaneous synthesis, dietary intake and absorption, hepatic 25-hydroxylation, renal activation, storage in adipose tissue, and degradation; therefore, several pathways could potentially contribute to an observed association between urinary glyphosate and serum 25(OH)D. Vitamin D hydroxylation is mediated in part by cytochrome P450 enzymes, particularly CYP2R1 and CYP27A1, which catalyze the conversion of cholecalciferol to 25(OH)D in the liver. Studies in rodents exposed to Roundup^®^ have demonstrated a more than 50% reduction in hepatic CYP enzyme levels compared with controls [22], an inhibition potentially attributed to glyphosate’s ability to chelate manganese, an essential cofactor for CYP enzyme function [49]. Additionally, glyphosate-induced oxidative stress and mitochondrial dysfunction may impair CYP enzyme function, supported by a similar association study using the NHANES database [50]. However, these findings do not establish that environmental glyphosate exposure impairs vitamin D metabolism in humans.

Other possible pathways include intestinal and endocrine homeostasis. Glyphosate was also demonstrated to disrupt gut microbiota composition, reducing *Bifidobacterium* spp., *Ruminococcus* spp., and *Lactobacillus* spp., which could affect intestinal barrier function and nutrient absorption [51,52,53]. Finally, glyphosate was shown to alter serum phosphate, calcium, and alkaline phosphatase levels, which may secondarily impair the vitamin D-parathyroid hormone axis [54]. Glyphosate may also exert endocrine-disrupting effects through inhibiting aromatase activity by up to 30%, which may contribute to vitamin D deficiency and reduced VDR expression [55]. A recent study demonstrated that high glyphosate exposure is associated with decreased estradiol, estrone, and estrone sulfate levels [56]. Conversely, vitamin D deficiency may itself contribute to endocrine and reproductive dysfunction, as vitamin D signaling is involved in steroidogenesis [57]. Thus, glyphosate-related endocrine disruption and low vitamin D status may potentially coexist within a bidirectional network of endocrine and reproductive effects; this possibility requires prospective investigation. Similarly, possible effects involving intestinal absorption, gut microbiota, vitamin D-binding protein, vitamin D receptor signaling, endocrine pathways, or renal handling remain speculative and require direct mechanistic investigation.

The increased association between serum 25(OH)D and dietary vitamin D intake in high-glyphosate-exposure individuals represents a novel finding of the current study and suggests a specific pattern of glyphosate-induced metabolic dysfunction—possibly impaired endogenous production with preserved capacity for dietary absorption. The non-linear dose-response pattern observed is consistent with toxicological relationships [58]. This pattern may reflect a threshold effect wherein moderate-to-high glyphosate exposure potentially induces maximal suppression of endogenous vitamin D production. Given that glyphosate was detected in the urine of approximately 70% of the U.S. population [59] and the prevalence of vitamin D insufficiency is high [60], the observed association may have potential public health relevance.

Several limitations warrant acknowledgment. First, this analysis is observational and cross-sectional in design, precluding causal inference. Direct experimental studies, including transporter- and vitamin D-metabolism assays, as well as longitudinal human studies with repeated exposure assessment, are required to evaluate these hypotheses. Although the multivariable adjustment included major confounders, unmeasured or residual confounding cannot be excluded. Geographic location, urban or rural residence, regional climate, ambient UVB exposure, time spent outdoors, vegetable consumption, and broader dietary patterns may affect both urinary glyphosate concentrations and vitamin D status. Future studies should incorporate geocoded environmental information, direct measures of UVB exposure, and detailed dietary-pattern and food-source data. Second, urinary glyphosate concentration reflects recent exposure over approximately 24–48 h prior to sample collection, which is an inherent constraint of all urinary biomarkers. No validated method yet exists for quantifying chronic cumulative glyphosate exposure at population scale. As a result, the extent to which individuals with sustained, low-level dietary exposure differ in their health outcomes from those with intermittent peak exposures remains an open question, not a limitation unique to this study. The reliance on a single spot urine sample is consistent with standard practice in large cohort studies and, while it introduces non-differential measurement error, this would most likely attenuate associations toward the null—meaning this measurement error may have biased the association toward or away from the null; its direction and magnitude cannot be determined from the present data. Third, the study population is limited to participants in NHANES cycles with both urinary glyphosate and serum 25(OH)D measurements, which may introduce selection bias. However, NHANES employs probability-based sampling designed to represent the non-institutionalized U.S. population, and this limitation is unlikely to substantially distort estimates of the glyphosate-vitamin D association. Finally, biomarkers of the proposed pathways—including CYP activity, manganese status, oxidative stress, mitochondrial function, gut microbiota, calcium/phosphate regulation, endocrine function, vitamin D-binding protein, vitamin D receptor signaling, and vitamin D absorption or transport—were not measured; consequently, the study cannot evaluate or distinguish these potential mechanisms.

## 5. Conclusions

Environmental glyphosate exposure, expressed as urinary glyphosate concentration, was found to be inversely associated with serum 25(OH)D concentrations in the U.S. adult population, with the relationship being obscured by dominant confounders including age, sex, BMI, socioeconomic status, and season. Based on the stratified analysis, individuals with high urinary glyphosate concentrations showed a stronger association between total vitamin D intake and serum 25(OH)D levels. These findings do not establish causality or identify the pathway responsible for the association but suggest that glyphosate exposure may represent an unrecognized risk factor for vitamin D deficiency. Prospective studies with repeated glyphosate and vitamin D measurements, together with experimental studies of vitamin D absorption, transport, hydroxylation, and signaling, are needed to assess temporality and mechanism.

## Figures and Tables

**Table 1:** Demographic and anthropometric characteristics of participants in NHANES 2013–2018.

Variable (*N* = 222,906,724)	Tertile of Urinary Glyphosate Concentration			
	Q1 Low	Q2 Medium	Q3 High	*p*-Trend
Age (years)	46.3 ± 16.7	48.2 ± 16.9	49.6 ± 17.6	<0.0001
Male (%)	46.1	46.8	52.9	0.0003
Non-Hispanic Black (%)	9.3	11.5	12.6	0.0042
Non-Hispanic White (%)	61.9	65.2	65.7	0.0325
Hispanic incl Mexican American (%)	16.5	15.7	13.5	0.0273
Other race (%)	12.3	7.6	8.2	0.0001
College and above (%)	64.9	65.9	62.9	0.2724
BMI (kg/m^2^)	28.7 ± 6.8	29.7 ± 7.2	29.9 ± 7.4	<0.0001
Income-to-poverty	3.2 ± 1.6	3.0 ± 1.7	3.0 ± 1.7	0.0002
Smoking: current smoker (%)	41.3	43.1	43.8	0.1587
Physical activity: active (%)	56.4	54.5	53.9	0.1783
Alcohol consumption: abstainers/light consumer (%)	47.4	44.7	47.2	0.8466
Renal function eGFR (ml/min)	99.0 ± 19.6	94.4 ± 21.5	93.9 ± 21.5	<0.0001
Vitamin D dietary intake (μg/day)	4.5 ± 4.3	4.8 ± 5.8	4.4 ± 4.3	0.7116
Vitamin D supplement users (%)	35.6	34.5	32.7	0.0976
Vitamin D supplements (μg/day) †	59.7 ± 153.3	48.3 ± 111.5	46.6 ± 73.1	0.0911
Serum 25(OH)D (nmol/L)	72.9 ± 30.0	71.9 ± 30.0	69.2 ± 28.9	0.0009
Urinary glyphosate concentration (range, µg/L)	0.071–0.216 *	0.217–0.455	0.456–8.21	

Note: Continuous variables presented as weighted Mean ± SD; Categorical variables as weighted %. *p*-trend calculated using linear regression (continuous) or chi-square test (categorical). * In 272 urine samples, the glyphosate concentration was below the limit of detection (0.1 µg/L). † Among supplement users only.

**Table 2:** Weighted regression models for the association between serum 25(OH)D levels and glyphosate exposure.

Model (*n*)	Adjustment Set	Glyphosate Association	Model Fit
1: Unadjusted (*n* = 4284)	None	β = −3.537; *p* = 0.035	R^2^ = 0.002, AIC = 41,209.90
2: Demographics (*n* = 3997)	Age, sex, race, BMI, season, total vitamin D intake	β = −4.793; *p* = 0.0025	R^2^ = 0.2773, AIC = 37,244.98
3: Lifestyle (*n* = 3645)	Model 2 + income, education, physical activity, smoking, alcohol	β= −4.208; *p* = 0.009	R^2^: 0.3034 AIC: 33,833.58
4: Fully adjusted (*n* = 3645)	Model 3 + CKD stages	β= −4.231; *p* = 0.0085	R^2^: 0.3083 AIC: 33,813.55
5: Fully adjusted with urinary creatinine (*n* = 3645)	Model 3 + urinary creatinine	β= − 3.361; *p* = 0.056	R^2^: 0.3037 AIC: 33,835.01

**Table 3:** Results of the fully adjusted survey-weighted regression model with design-based standard errors for the association between serum 25(OH)D levels and demographic, socioeconomic, lifestyle, and environmental determinants (*n* = 3645).

Covariate	Category/Variable	β Coefficient	Standard Error	t-Value	*p*-Value
Age	40–59	8.268	1.384	5.974	<0.001
	60+	16.037	1.787	8.976	<0.001
Sex	Male	−5.755	0.955	−6.024	<0.001
Race	Non-Hispanic Black	−9.405	1.399	−6.723	<0.001
	Non-Hispanic White	12.227	1.391	8.788	<0.001
	Other race	0.117	2.015	0.058	0.954
BMI	Overweight	−2.493	1.240	−2.010	0.056
	Obesity	−8.665	1.210	−7.159	<0.001
Season	Winter	−4.733	1.302	−3.634	0.0014
Education	High school or less	−0.354	1.189	−0.297	0.769
Income-to-poverty	1.31–3.50	−4.082	1.109	−3.681	0.0012
	>3.50	3.964	1.270	3.121	0.0048
Physical activity	Inactive	−4.014	0.928	−4.328	<0.001
Smoking status	Current smoker	−1.133	1.142	−0.993	0.3312
Alcohol consumption	Moderate/Heavy	0.547	1.683	0.325	0.748
Total vitamin D intake	Standardized intake	6.775	2.561	2.645	0.0145
Glyphosate exposure	Log-transformed urinary Glyphosate	−4.231	1.470	−2.879	0.0085

## Data Availability

The data that support the findings of this study are available in the NHANES database at https://wwwn.cdc.gov/nchs/nhanes/search/datapage.aspx (accessed on 27 November 2025). The analytic code used for data processing and statistical modeling can be obtained from the corresponding author upon reasonable request.

## Supplementary Materials

The following supporting information can be downloaded at: https://www.mdpi.com/article/10.3390/biology15171510/s1, Table S1: Multicollinearity diagnostics: weighted variance inflation factors (VIFs) for all categorical variables included in the fully adjusted survey-weighted regression model; Table S2: Sensitivity analysis using log-transformed serum 25(OH)D values to assess robustness of the association between glyphosate exposure and serum 25(OH)D; Table S3: Fully adjusted association between vitamin D intake and serum 25(OH)D by glyphosate exposure tertile.

## Abbreviations

The following abbreviations are used in this manuscript:

NHANES	National Health and Nutrition Examination Survey
CKD	Chronic kidney disease
25(OH)D	25-hydroxy vitamin D
CI	confidence intervals
BMI	Body Mass Index
AIC	Akaike Information Criterion
VIF	variance inflation factors
eGFR	Estimated glomerular filtration rate

## References

[1] Benbrook C.M. (2016). Trends in glyphosate herbicide use in the United States and globally. Environ. Sci. Eur..

[2] Madani N.A., Carpenter D.O. (2022). Effects of glyphosate and glyphosate-based herbicides like Roundup^TM^ on the mammalian nervous system: A review. Environ. Res..

[3] Li W., Lei D., Huang G., Tang N., Lu P., Jiang L., Lv J., Lin Y., Xu F., Qin Y. (2023). Association of glyphosate exposure with multiple adverse outcomes and potential mediators. Chemosphere.

[4] Liang Z., Sun X., Guo R., Wang H., Tian Y., Wang Y., Liu Y., Liu S. (2024). Association between glyphosate exposure and osteoarthritis in US adults: Especially in people who are obese and inactive in leisure time physical activity. Sci. Total Environ..

[5] Mazuryk J., Klepacka K., Kutner W., Sharma P.S. (2024). Glyphosate: Hepatotoxicity, Nephrotoxicity, Hemotoxicity, Carcinogenicity, and Clinical Cases of Endocrine, Reproductive, Cardiovascular, and Pulmonary System Intoxication. ACS Pharmacol. Transl. Sci..

[6] Tizhe E.V., Igbokwe I.O., Njokwu C.O.-I., Fatihu M.Y., Tizhe U.D., Ibrahim N.D.-G. (2024). Effect of Zinc Supplementation on Altered Calcium Homeostasis, Parathyroid Gland, Bone, and Skeletal Muscle Histology Induced by Subchronic Oral Exposure to Glyphosate-Based Herbicide (GOBARA^®^) in Wistar Rats. Clin. Pathol..

[7] Muñoz J.P., Silva-Pavez E., Carrillo-Beltrán D., Calaf G.M. (2023). Occurrence and exposure assessment of glyphosate in the environment and its impact on human beings. Environ. Res..

[8] Conrad A., Schröter-Kermani C., Hoppe H.-W., Rüther M., Pieper S., Kolossa-Gehring M. (2017). Glyphosate in German adults—Time trend (2001 to 2015) of human exposure to a widely used herbicide. Int. J. Hyg. Environ. Health.

[9] Wimalawansa S.J. (2024). Physiology of Vitamin D—Focusing on Disease Prevention. Nutrients.

[10] Cui X., Eyles D.W. (2022). Vitamin D and the Central Nervous System: Causative and Preventative Mechanisms in Brain Disorders. Nutrients.

[11] Gáll Z., Székely O. (2021). Role of vitamin d in cognitive dysfunction: New molecular concepts and discrepancies between animal and human findings. Nutrients.

[12] Wimalawansa S.J. (2023). Infections and Autoimmunity—The Immune System and Vitamin D: A Systematic Review. Nutrients.

[13] Pál É., Ungvári Z., Benyó Z., Várbíró S. (2023). Role of Vitamin D Deficiency in the Pathogenesis of Cardiovascular and Cerebrovascular Diseases. Nutrients.

[14] Amirkhizi F., Khademi Z., Hamedi−Shahraki S., Rahimlou M. (2023). Vitamin D insufficiency and its association with adipokines and atherogenic indices in patients with metabolic syndrome: A case-control study. Front. Endocrinol..

[15] Lai Y.-C., Chen Y.-H., Liang F.-W., Wu Y.-C., Wang J.-J., Lim S.-W., Ho C.-H. (2023). Determinants of cancer incidence and mortality among people with vitamin D deficiency: An epidemiology study using a real-world population database. Front. Nutr..

[16] Seiler N., Tsiglopoulos J., Keem M., Das S., Waterdrinker A. (2022). Prevalence of vitamin D deficiency among psychiatric inpatients: A systematic review. Int. J. Psychiatry Clin. Pract..

[17] Liu Y., Gong C., Li J., Ning X., Zeng P., Wang L., Lian B., Liu J., Fang L., Guo J. (2024). Vitamin D content and prevalence of vitamin D deficiency in patients with epilepsy: A systematic review and meta-analysis. Front. Nutr..

[18] Mousavi S.E., Amini H., Heydarpour P., Amini Chermahini F., Godderis L. (2019). Air pollution, environmental chemicals, and smoking may trigger vitamin D deficiency: Evidence and potential mechanisms. Environ. Int..

[19] Bikle D.D. (2014). Vitamin D Metabolism, Mechanism of Action, and Clinical Applications. Chem. Biol..

[20] Jones G., Prosser D.E., Kaufmann M. (2014). Cytochrome P450-mediated metabolism of vitamin D. J. Lipid Res..

[21] Slominski A.T., Kim T.-K., Li W., Yi A.-K., Postlethwaite A., Tuckey R.C. (2014). The role of CYP11A1 in the production of vitamin D metabolites and their role in the regulation of epidermal functions. J. Steroid Biochem. Mol. Biol..

[22] Larsen K., Najle R., Lifschitz A., Maté M.L., Lanusse C., Virkel G.L. (2014). Effects of Sublethal Exposure to a Glyphosate-Based Herbicide Formulation on Metabolic Activities of Different Xenobiotic-Metabolizing Enzymes in Rats. Int. J. Toxicol..

[23] Estienne A., Fréville M., Bernardi O., Ramé C., Calandreau L., Cornilleau F., Ganier P., Chahnamian M., Froment P., Dupont J. (2023). Chronic dietary exposure to a glyphosate-based herbicide in broiler hens has long-term impacts on the progeny metabolism. Poult. Sci..

[24] Hamad N., Sharkawy A.A., Ibrahim N., Osman A.A.R., Aljohani A.S.M., Kamaly H.F. (2025). Aloe Vera alleviates glyphosate-based herbicide hepatotoxicity in rats via anti-inflammatory and antioxidant actions. Front. Vet. Sci..

[25] Vardakas P., Veskoukis A.S., Rossiou D., Gournikis C., Kapetanopoulou T., Karzi V., Docea A.O., Tsatsakis A., Kouretas D. (2022). A Mixture of Endocrine Disruptors and the Pesticide Roundup^®^ Induce Oxidative Stress in Rabbit Liver When Administered under the Long-Term Low-Dose Regimen: Reinforcing the Notion of Real-Life Risk Simulation. Toxics.

[26] Zheng T., Jia R., Cao L., Du J., Gu Z., He Q., Xu P., Yin G. (2021). Effects of chronic glyphosate exposure on antioxdative status, metabolism and immune response in tilapia (GIFT, *Oreochromis niloticus*). Comp. Biochem. Physiol. C Toxicol. Pharmacol..

[27] Yan B., Sun Y., Fu K., Zhang Y., Lei L., Men J., Guo Y., Wu S., Han J., Zhou B. (2023). Effects of glyphosate exposure on gut–liver axis: Metabolomic and mechanistic analysis in grass carp (*Ctenopharyngodon idellus*). Sci. Total Environ..

[28] Yang J.-H., Lee Y.-M., Bae S.-G., Jacobs D.R., Lee D.-H. (2012). Associations between Organochlorine Pesticides and Vitamin D Deficiency in the U.S. Population. PLoS ONE.

[29] Brennan E., Butler A.E., Nandakumar M., Drage D.S., Sathyapalan T., Atkin S.L. (2023). Association between Organochlorine Pesticides and Vitamin D in Female Subjects. Biomedicines.

[30] Feng X., Wang M., Wang Y., Liang R., Yan C. (2025). Associations between environmental glyphosate exposure and glucose homeostasis indices in US general adults: A national population-based cross-sectional study. Sci. Rep..

[31] Hsiao C.C., Yang A.-M., Wang C., Lin C.-Y. (2023). Association between glyphosate exposure and cognitive function, depression, and neurological diseases in a representative sample of US adults: NHANES 2013–2014 analysis. Environ. Res..

[32] Schütze A., Morales-Agudelo P., Vidal M., Calafat A.M., Ospina M. (2021). Quantification of glyphosate and other organophosphorus compounds in human urine via ion chromatography isotope dilution tandem mass spectrometry. Chemosphere.

[33] Barr D.B., Wilder L.C., Caudill S.P., Gonzalez A.J., Needham L.L., Pirkle J.L. (2005). Urinary Creatinine Concentrations in the U.S. Population: Implications for Urinary Biologic Monitoring Measurements. Environ. Health Perspect..

[34] O’Brien K.M., Upson K., Buckley J.P. (2017). Lipid and Creatinine Adjustment to Evaluate Health Effects of Environmental Exposures. Curr. Environ. Health Rep..

[35] Pludowski P., Holick M.F., Grant W.B., Konstantynowicz J., Mascarenhas M.R., Haq A., Povoroznyuk V., Balatska N., Barbosa A.P., Karonova T. (2018). Vitamin D supplementation guidelines. J. Steroid Biochem. Mol. Biol..

[36] Couch C.A., Williams A.M., Stierman B., Storandt R., Leachman J., Mishra S., Juan W., Gahche J.J., Wambogo E., Mineva E. (2026). Trends in vitamin D status in the United States, 2007–2023: A cross-sectional analysis of data from the National Health and Nutrition Examination Survey. Am. J. Clin. Nutr..

[37] Wan Z., Guo J., Pan A., Chen C., Liu L., Liu G. (2021). Association of Serum 25-Hydroxyvitamin D Concentrations with All-Cause and Cause-Specific Mortality Among Individuals with Diabetes. Diabetes Care.

[38] Yu G., Lin Y., Dai H., Xu J., Liu J. (2023). Association between serum 25-hydroxyvitamin D and osteoarthritis: A national population-based analysis of NHANES 2001–2018. Front. Nutr..

[39] Liu X., Baylin A., Levy P.D. (2018). Vitamin D deficiency and insufficiency among US adults: Prevalence, predictors and clinical implications. Br. J. Nutr..

[40] Cui A., Xiao P., Ma Y., Fan Z., Zhou F., Zheng J., Zhang L. (2022). Prevalence, trend, and predictor analyses of vitamin D deficiency in the US population, 2001–2018. Front. Nutr..

[41] Thuesen B., Husemoen L., Fenger M., Jakobsen J., Schwarz P., Toft U., Ovesen L., Jørgensen T., Linneberg A. (2012). Determinants of vitamin D status in a general population of Danish adults. Bone.

[42] Lips P., van Schoor N.M., de Jongh R.T. (2014). Diet, sun, and lifestyle as determinants of vitamin D status. Ann. N. Y. Acad. Sci..

[43] Tønnesen R., Hovind P.H., Jensen L.T., Schwarz P. (2016). Determinants of vitamin D status in young adults: Influence of lifestyle, sociodemographic and anthropometric factors. BMC Public Health.

[44] Morales-Villar A.B., Maldonado-Hernández J., Eduardo Álvarez-Licona N., Piña-Aguero M.I., Villalpando-Hernández S., Robledo-Pérez R.M., Díaz-Rangel I., Barbosa-Cortés M.d.L., Núñez-García B.-A. (2024). Determinants of Vitamin D Status in Healthy Young Adults from Mexico City. Arch. Med. Res..

[45] Park J., Ryu S.-Y., Han M., Choi S.-W. (2016). The Association of Vitamin D with Estimated Glomerular Filtration Rate and Albuminuria: 5th Korean National Health and Nutritional Examination Survey 2011–2012. J. Ren. Nutr..

[46] Hsu S., Zelnick L.R., Lin Y.S., Best C.M., Kestenbaum B., Thummel K.E., Rose L.M., Hoofnagle A.N., de Boer I.H. (2021). Differences in 25-Hydroxyvitamin D Clearance by eGFR and Race: A Pharmacokinetic Study. J. Am. Soc. Nephrol..

[47] Yang L., Zhao H., Liu K., Wang Y., Liu Q., Sun T., Chen S., Ren L. (2021). Smoking behavior and circulating vitamin D levels in adults: A meta-analysis. Food Sci. Nutr..

[48] Jiang C.Q., Chan Y.H., Xu L., Jin Y.L., Zhu T., Zhang W.S., Cheng K.K., Lam T.H. (2016). Smoking and serum vitamin D in older Chinese people: Cross-sectional analysis based on the Guangzhou Biobank Cohort Study. BMJ Open.

[49] Samsel A., Seneff S. (2015). Glyphosate, pathways to modern diseases III: Manganese, neurological diseases, and associated pathologies. Surg. Neurol. Int..

[50] Fang Y.-W., Lin H.-C., Wang C., Lin C.-Y. (2025). Glyphosate Exposure, Oxidative Stress, Mitochondrial Dysfunction, and Mortality Risk in US Adults: Insights from the National Health and Nutrition Examination Survey. Toxics.

[51] Lehman P.C., Cady N., Ghimire S., Shahi S.K., Shrode R.L., Lehmler H.-J., Mangalam A.K. (2023). Low-dose glyphosate exposure alters gut microbiota composition and modulates gut homeostasis. Environ. Toxicol. Pharmacol..

[52] Cáceres-Chacón M., Martínez-Guzmán O., Haddock-Martínez H.A., Figueroa-Pérez A., Rodríguez-Rosado S., Suárez-Pérez J., Ramos-Sánchez R.Y., Godoy-Vitorino F., Sierra-Mercado D. (2025). Exposure to the herbicide glyphosate leads to inappropriate threat responses and alters gut microbial composition. Front. Toxicol..

[53] Aitbali Y., Ba-M’hamed S., Elhidar N., Nafis A., Soraa N., Bennis M. (2018). Glyphosate based-herbicide exposure affects gut microbiota, anxiety and depression-like behaviors in mice. Neurotoxicol. Teratol..

[54] Mertens M., Höss S., Neumann G., Afzal J., Reichenbecher W. (2018). Glyphosate, a chelating agent—Relevant for ecological risk assessment?. Environ. Sci. Pollut. Res..

[55] Zhang C., Schilirò T., Gea M., Bianchi S., Spinello A., Magistrato A., Gilardi G., Di Nardo G. (2020). Molecular Basis for Endocrine Disruption by Pesticides Targeting Aromatase and Estrogen Receptor. Int. J. Environ. Res. Public Health.

[56] Wu W.-Y., Wang D.-S., Lin H.-C., Wang C., Lin C.-Y. (2025). Urinary Glyphosate Concentrations and Serum Sex Hormones in a Nationally Representative U.S. Sample: NHANES 2017–2018. Life.

[57] Kolcsár M., Berecki B., Gáll Z. (2023). Relationship between Serum 25-Hydroxyvitamin D Levels and Hormonal Status in Infertile Women: A Retrospective Study. Diagnostics.

[58] Li L., Li D. (2021). Inter-Individual Variability and Non-linear Dose-Response Relationship in Assessing Human Health Impact From Chemicals in LCA: Addressing Uncertainties in Exposure and Toxicological Susceptibility. Front. Sustain..

[59] Ospina M., Schütze A., Morales-Agudelo P., Vidal M., Wong L.-Y., Calafat A.M. (2022). Exposure to glyphosate in the United States: Data from the 2013–2014 National Health and Nutrition Examination Survey. Environ. Int..

[60] Amrein K., Scherkl M., Hoffmann M., Neuwersch-Sommeregger S., Köstenberger M., Tmava Berisha A., Martucci G., Pilz S., Malle O. (2020). Vitamin D deficiency 2.0: An update on the current status worldwide. Eur. J. Clin. Nutr..

