# Supplementary material for: Environmental Glyphosate Exposure Is Associated with Lower Serum 25-Hydroxyvitamin D Concentrations: A Cross-Sectional Analysis of NHANES 2013–2018
Source: Biology (Basel). 2026 Sep 3;15(17):1510. doi: 10.3390/biology15171510 (PMC13564523; doi:10.3390/biology15171510)
Supplement: Supplementary file 1 [file biology-15-01510-s001.zip › biology-4527046-supplementary.pdf]

Environmental glyphosate exposure is associated with lower serum 25-hydroxyvitamin D concentrations: a cross-sectional analysis of NHANES 2013–2018

Zsolt Gáll[1,2], Lénárd Farczádi[3], Melinda Kolcsar[1]

[1]Department of Pharmacology and Clinical Pharmacy, George Emil Palade University of Medicine, Pharmacy, Science and Technology of Targu Mures, Gheorghe Marinescu street, no. 38, 540142 Targu Mures, Romania

[2]Scidata Research and Training Ltd., 547367 Targu Mures, Romania

[3]Chromatography and Mass Spectrometry Laboratory, Center for Advanced Medical and Pharmaceutical Research, George Emil Palade University of Medicine, Pharmacy, Science, and Technology of Targu Mures, 540142 Targu Mures, Romania

Contact: zsolt.gall@umfst.ro

Table S1. Multicollinearity diagnostics: weighted variance inflation factors (VIFs) for all categorical variables included in the fully adjusted survey-weighted regression model.

**Covariate**	**VIF**
Age	2.15
Gender	2.10
Race/ethnicity	3.16
Season	1.85
Education	1.87
Body-mass-index	2.32
Income-to-poverty	2.78
Smoking status	1.96
Alcohol consumption	1.73
Physical activity	2.21
Kidney function	1.10

Table S2. Sensitivity analysis using log-transformed serum 25(OH)D values to assess robustness of the association between glyphosate exposure and serum 25(OH)D.

Model	Outcome scale	N	β for glyphosate (95% CI)	p-value	R^2	AIC*
CKD stage adjustment	25(OH)D (nmol/L)	3645	−4.25 (−7.31, −1.19)	0.0086	0.308	33,807
CKD stage adjustment	Log_{10}[25(OH)D]	3645	−0.029 (−0.048, − 0.011)	0.0034	0.312	−3039
Urinary creatinine adjustment	25(OH)D (nmol/L)	3645	−3.36 (−6.83, 0.098)	0.056	0.304	33,835
Urinary creatinine adjustment	Log_{10}[25(OH)D]	3645	−0.025 (−0.047, −0.004)	0.023	0.309	-3015

All models were survey-weighted WLS and adjust for age, sex, race/ethnicity, body mass index, season, education, income, physical activity, smoking, alcohol intake, and kidney function.

* Because the two models are fitted to differently transformed outcomes, AIC values are not strictly comparable across specifications and are reported here for completeness only.

Table S3. Fully adjusted association between vitamin D intake and serum 25(OH)D by glyphosate exposure tertile

Glyphosate Tertile	N	Vitamin D Intake β	95% CI	p-value	R^2	Adj R^2
Low	1,216	7.41	(1.48, 13.34)	0.0165	0.335	0.324
Medium	1,214	5.499	(-0.33, 11.33)	0.0634	0.313	0.302
High	1,215	12.88	(3.934, 21.83)	0.0066	0.326	0.314
